# Expression of LamB Vaccine Antigen in *Wolffia globosa* (Duck Weed) Against Fish Vibriosis

**DOI:** 10.3389/fimmu.2020.01857

**Published:** 2020-08-20

**Authors:** P. P. M. Heenatigala, Zuoliang Sun, Jingjing Yang, Xuyao Zhao, Hongwei Hou

**Affiliations:** ^1^University of Chinese Academy of Sciences, Beijing, China; ^2^The State Key Laboratory of Freshwater Ecology and Biotechnology, The Key Laboratory of Aquatic Biodiversity and Conservation of Chinese Academy of Sciences, Institute of Hydrobiology, Chinese Academy of Sciences, Wuhan, China; ^3^Inland Aquatic Resources and Aquaculture Division (IARAD), National Aquatic Resources Research and Development Agency (NARA), Colombo, Sri Lanka

**Keywords:** vibriosis, *Wolffia globosa*, LamB, edible vaccine, recombinant protein, oral immunization

## Abstract

Vibriosis is a commonly found bacterial disease identified among fish and shellfish cultured in saline waters. A multitude of *Vibrio* species have been identified as the causative agents. LamB, a member of outer membrane protein (OMPs) family of these bacteria is conserved among all *Vibrio* species and has been identified as an efficient vaccine candidate against vibriosis. Rootless duckweed (*Wolffia*) is a tiny, edible aquatic plant possessing characteristics suitable for the utilization as a bioreactor. Thus, we attempted to express a protective edible vaccine antigen against fish vibriosis in nuclear-transformed *Wolffia*. We amplified *LamB* gene from virulent *Vibrio alginolyticus* and it was modified to maximize the protein expression level and translocate the protein to the endoplasmic reticulum (ER) in plants. It was cloned into binary vector pMYC under the control of CaMV 35S promoter and introduced into *Wolffia globosa* by *Agrobacterium*-mediated transformation. Integration and expression of the *LamB* gene was confirmed by genomic PCR and RT-PCR. Western blot analysis revealed accumulation of the LamB protein in 8 transgenic lines. The cross-protective property of transgenic *Wolffia* was evaluated by orally vaccinating zebrafish through feeding fresh transgenic *Wolffia* and subsequently challenging with virulent *V. alginolyticus*. High relative percent survival (RPS) of the vaccinated fish (63.3%) confirmed that fish immunized with transgenic *Wolffia* were well-protected from *Vibrio* infection. These findings suggest that *Wolffia* expressed LamB could serve as an edible plant-based candidate vaccine model for fish vibriosis and feasibility of utilizing *Wolffia* as bioreactor to produce edible vaccines.

## Introduction

Vibriosis is a serious disease commonly identified among fish and shellfish aquaculture and has become a major limiting factor in the aquaculture industry worldwide. A group of Gram-negative bacteria inhabiting in saline waters belonging to the genus *Vibrio* has been identified as the causative agent (1). Massive (mis) use of antibacterials to control vibriosis has resulted in severe environmental as well as health concerns (2). Thus, development of vaccination strategies against vibriosis will be an effective solution for the management of vibriosis in aquaculture. Most vaccines currently used (especially for viral diseases) come from lab cultured pathogens through attenuation or inactivation, bringing potential risk of residual pathogenic activity. In this regard, recombinant proteins expressed in plant bioreactors are safer and more reliable, as they contain specific components of pathogens with the immunological properties of the original pathogen but not its pathogenic properties (3, 4).

Among fish vaccine delivery techniques, oral routes (oral immunization) would be an attractive alternative, as it is simple, cheap, and ideal for mass administration to fish of all sizes without causing a stress. However, oral immunization is a multifaceted process, depending on multiple cellular and molecular mechanisms. As teleost, fish lack Peyer's patches and antigen-transporting M cells, which are important to initiate the gut immune responses but their second segment of the gut has been identified as the main site of antigen uptake (5, 6). For that, many lymphoid cells and macrophages are diffusely present between the epithelial cells and in the lamina propria enterocytes of the hind gut (6, 7). Thus, as teleost fish intestine can easily be exploited for oral vaccination strategies.

Oral vaccination of fish has become less effective due to digestive degradations of antigens in the acidic environment of the foregut, before it reaches the immune responsive areas of the hind gut (8). Thus, while developing edible vaccines, special concern should be given to protect antigen from the harsh gastric environment to ensure antigen uptake in the second gut segment of fish. Protective antigens expressed in transgenic plants are the ideal solution for such issues. Thick, rigid cell walls of the plants encapsulate the antigenic proteins, thus protecting them well from the acidic environment of the stomach. They act as vehicles to orally deliver protective antigens (9) to get through the acidic environment of the foregut without intestinal degradation. Thus, the antigen can reach the second gut in the intestine safely in sufficient quantities and successful oral vaccination can be achieved.

Outer membrane proteins (OMPs) are unique components reside in the outer membrane of Gram-negative bacteria and responsible for maintaining the integrity and selective permeability of the bacterial outer membrane (10). As they are being localized at the bacterial cell surfaces, OMPs of Gram-negative bacteria could be efficiently recognized as foreign substances by immunological defense systems of hosts. Thus, OMPs of Gram-negative bacteria have been identified as highly immunogenic components due to their exposed epitopes as well as being conserved in nature (11). LamB proteins are a family of OMPs identified in vibrios and Lun et al. (12) has reported that it can be used as a broad cross-protective vaccine candidate against vibriosis.

*Wolffia*, the rootless duckweed is the smallest member of the duckweed family as well as in the plant kingdom (13). Due to its prominent asexual propagation, rootless characteristics and extremely reduced size, it has become an attractive, highly efficient bio-manufacturing platform for foreign protein production and makes it a more suitable candidate for submerged cultivation in bio-fermenters (14). Thus, high biomass of genetically homogeneous *Wolffia* populations can be easily obtained within the compact environment on artificial media. As it is an edible plant, the proteins in transgenic *Wolffia* can be directly consumed without extraction and/or purification of the active constituent. This is one of the main advantages identified in this system and makes *Wolffia* a more attractive member among the duckweed family, especially in edible vaccine research.

Lack of an efficient gene transformation protocol for *Wolffia* has limited the utilization of this valuable plant as a bio-manufacturing platform. To fulfill this gap in our previous work, we developed an efficient gene transformation protocol for *W. globosa*, the *Wolffia* species available in China (15). As the second step, here we focused on exploring the feasibility to express an edible protective vaccine antigen LamB for fish vibriosis in *W. globosa*.

## Materials and Methods

### Amplification of Gene of Interest

LamB outer membrane protein of *Vibrio* bacteria was selected as the antigenic protein to express in *W. globosa*. The pathogenic *Vibrio alginolyticus* ECGY0608 (kindly given by Professor Ai-hua Li, Institute of Hydrobiology, Chinese Academy of Sciences) isolated from diseased fish was used to amplify the *LamB* gene. Genomic DNA of *V. alginolyticus* was extracted from overnight cultures in the TSA medium with a TIANamp Bacteria DNA kit (Cat.−DP 302, Tian GEN Biotech Beijin Co. LTD) according to the manufacturer's protocol. DNA extracts obtained were used as templates to amplify the *LamB* gene using degenerate primers. The primer sequences were lamB-f: 5-ATG AAA AAA GTA AGT SNY ATT GCA G-3 and LamB-r: 5-TTA CCA CCA AGC TTC NRC TTG-3 (12). PCR was carried out in a thermal cycler (Eppendorff, USA) with the following set up: Initial denaturation for 5 min at 95°C, Then 30 cycles were run with conditions: 95°C for 30 s, 48°C for 45 s, and 72°C for 1 min and final cycle of 72°C for 7 min. The PCR product was analyzed by electrophoresis on a 1.2% agarose gel, and purified with Easy Pure PCR purification kit (TRANS Gen Biotech). The amplified DNA fragment was cloned into the pGEM-T-Easy vector (Promega) and sequenced (http://www.tsingke.net).

### Database Searching and Bioinformatics Analysis

The identities of the nucleotide sequences were determined by comparing with known sequences in GenBank using the respective BLAST program available at NCBI (http://www.ncbi.nlm.nih.gov/).

### Designing and Construction of Plant Transformation Vector for *Agrobacterium*-Mediated Transformation of *W. globosa*

The *LamB* gene obtained was further modified to achieve higher protein expression, accumulation, and isolation. To enhance *Agrobacterium tumefaciens*-mediated stable gene expression in *W. globosa*, we designed the construct to express ER targeted LamB fusion protein under the control of cauliflower mosaic virus (CaMV) 35S promoter, a tCUP translational enhancer, PR1b signal peptide, and the nopaline synthase (nos) terminator in the plant expression vector pMYC.

**Figure 1:**
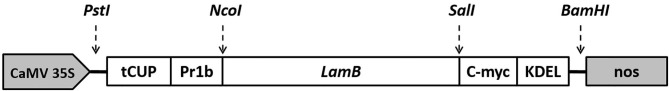
Schematic representation of T-DNA region of the expression vector (pMYC-*LamB*) construct used for *W. globosa* transformation. Transgene expression fragment (*LamB*) was placed under the control of the cauliflower mosaic virus 35S promoter, tCUP, translational enhancer from the tobacco cryptic upstream promoter; Pr1b, tobacco pathogenesis related 1b protein secretary signal peptide; C-myc, detection/purification tag; KDEL, endoplasmic reticulum retrieval tetrapeptide; nos, nopaline synthase transcription terminator.

Thus, tobacco cryptic constitutive promoter (tCUP) enhancer and pathogenesis-related protein 1b (PR1b) signal peptide were synthesized (http://www.tsingke.net) and spliced together with the 5′ end of the *LamB* sequence by overlap extension (16). MYC protein tag and KDEL retention signal peptide was incorporated to the 3′ end of *LamB* by newly designed primers. Specific restriction sites (*Pst1/Ncol1/Sal1/BamHI*) were designed from the cloned nucleotide sequence to amplify the encoding sequence for the mature LamB protein, and incorporated into the PCR during *LamB* gene modification. Primer sequences used to modify the *LamB* gene are shown in Table 1. Schematic representation of the expression cassette (modified *LamB* gene) is shown in Figure 1. It was sub-cloned into the pGEM-T-Easy vector (Promega) and the modifications were confirmed by sequencing (http://www.tsingke.net).

**Table 1:** Primers used to modify *LamB* gene.

**Primers**	**Sequence (5′- 3′)**	**Annealing temperature (°C)**
tCUP Pst2 (F)	TGCACTGCAGAATACTAGCCTATT	55.6
Prb1 Nocl (R)	TTACTTTTTTCATCCATGGGGCAGGGGAAG	
Lamb Modi (F)	CTTCCCGTGCCCCATGGATGAAAAAAGTAA	56
Lamb Corr (R)	TTCTTCAGAGATCAGTTTCTGTTCGTCGACCCACCAAG	
Lamb Modi (F)	CTTCCCGTGCCCCATGGATGAAAAAAGTAA	57
Lamb Modi (R)	CGCGGATCCGAGCTCATCCTTCAGATCTTCTTCAGA	
Overlap F—a	TGCACTGCAGAATACTAGCCTATTTTATTTCAA	58.7
Overlap R—a	CGCGGATCCGAGCTCAT	

Then the LamB expression cassette was cut out from the pGEM-T-Easy between cut sites *Pst1* and *BamHI* and using the same sites, cloned into pMYC binary vector under the control of the CaMV 35S promoter. Thus, the gene was located downstream from the 35S cauliflower mosaic virus promoter (CaMV35S) and upstream of the nopaline synthase 3′ UTR (NOSt) to give the binary vector pMYC-*LamB*. We confirmed the successful insertion of the LamB expression cassette into the binary vector pMYC-*LamB* by restriction enzyme digestion and sequencing (http://www.tsingke.net). The resulting binary vector pMYC-LamB was used for LamB protein expression in *W. globosa*, following its *Agrobacterium*-mediated transformation (15).

Thus, the construct (pMYC-*LamB*) was mobilized into the commercially available disarmed *Agrobacterium* strain EHA105 (http://www.transgen.com.cn/) by heat shock according to the manufacture's protocol and used for the transformation of *W. globosa*.

### Tissue Culture and *Agrobacterium-*Mediated Transformation of *W. globosa*

*W. globosa* fronds were collected from Wuhan Botanical Garden (RDSC Clone *W. globosa* 5563**)**, Chinese Academy of Sciences (CAS) (30.54° N and 114.42° E) at the city of Wuhan, Hubei province, China. Aseptic populations of *W. globosa* single clones were obtained in solid SH medium (17) with 2% sucrose and 0.6% agar and used as explants in this study. *W. globosa* clusters were induced by culturing explants in cluster induction medium for 4 months (18). Clusters obtained were used for the transformation experiments.

*Agrobacterium*-mediated nuclear transformation was performed as previously described (15). Briefly, *W. globosa* clusters were mixed with glass beads and bacterial suspension of *A. tumefaciens* (EHA105 harboring the pMYC-*LamB* construct) and subjected to vigorous shaking for 30 min to injure the clusters. Subsequently, these were blotted onto sterile filter paper, and co-cultivated for 72 h on antibiotic-free liquid SH medium. Then clusters were subjected to 2 week resting period and finally for 1 month selection. Plant selectable marker used in the construct was Hyg-R. Thus, Hygromycin B (Hyg) was used as the selection agent at the concentration of 5.0 mgl^−1^ to select transgenic explants as well as to obtain pure transgenic lines_._ All steps of transformation experiments were carried out at 25 ± 1°C under the white light of 85 μmol m^−2^ s^−1^, 16: 8 h light: dark photoperiod.

### Genomic Analysis of Transgene Integration

To confirm gene integration within the plant genome we first performed a PCR assay. Total genomic DNA from the putative transgenic and wild-type *W. globosa* explants was extracted using a plant genome extraction kit, NuClean PlantGen DNA Kit (CWBIO, China) (http://www.cwbiotech.com.cn/) according to the manufacture's protocol. Genomic DNA obtained was used as the template to amplify the modified *LamB* gene integrated. PCR analysis of putatively transgenic plants was performed using PCR primers, Overlap F—a and Overlap R—a (Table 1). Overlap PCR primers amplify a 1,548-bp fragment comprising the sequence of modified *Lam B* gene.

### RT-PCR Detection of the *LamB* Gene Integrated

Total RNA from wild and transformed *W. globosa* was extracted using the Trizol reagent (Invitrogen). cDNA was synthesized with 2 μg of total RNA using a Prime Script RT reagent Kit (Takara) according to the manufacture's protocol. RT-PCR for *LamB* was conducted with newly designed *LamB* gene specific primers (*LamB*-RT-F: 5′-GTTTCTTTCGCTTGGGTTCG-3′, *LamB*-RT-R: 5′-CATTACGCCGTTTTTCGCAT-3′). An *Actin* gene was used as the internal control. *Actin* gene was amplified using two degenerate primers, ActF: 5′-GTGYTKGAYTCTGGTGATGGTGT-3′ and ActR: 5′-ACCTTRATCTTCATGCTGCTSGG-3′ (15).

### Immunoblot Detection of LamB Protein in Transgenic *W. globosa*

Total soluble protein was extracted from transgenic and wild type *Wolffia* fronds. The fronds were ground in liquid nitrogen to a fine powder. 0.5 g powder was re-suspended in 4 volumes of extraction buffer [50 mM Tris–HCl, pH 8.0, 10 mM EDTA, pH 8.0, 10% (v/v) glycerol, 1% (w/v) SDS, 30 mM 2-mercaptoethanol, 4 μg/ml aprotinin, and 4 μg/ml leupeptin]. Total proteins were extracted for 20 min at 4°C and then centrifuged at 14,000× g for 10 min at 4°C. Supernatant was taken for further analysis. Protein extracts were quantified with the help of BCA protein assay kit (Beyotime, China, Cat. P0012) according to the manufacturer's instructions.

Protein extracts were subjected to fully automated capillary electrophoresis (CE) based Western blot assay to identify the recombinant LamB protein in transgenic *Wolffia*. Automated Western blot was performed by the Peggy Sue instrument, Protein Simple (Santa Clara, CA, USA). Biotinylated molecular weight ladder, streptavidin-HRP, DTT, fluorescent standards, luminol-S, peroxide, sample buffer, stacking matrix, separation matrix, running buffers, wash buffer, and the HRP-conjugated secondary antibody, were used according to the manufacture's protocol. Mouse anti-myc (QW–bio, China) was used as the primary antibody. “Virtual blot” electrophoretic images were generated using Compass Software (ProteinSimple). The kit also provides capillaries, antibody diluents, and sample loading plates.

### Fish Immunization

Healthy and *V. alginolyticus* infection-free four months old wild type zebrafish (AB strain) obtained from single parental stock were purchased from a fish farm/Institute of Hydrobiology (Wuhan, China) (mean weight 0.4 g; length 2–3 cm) and acclimatized for 2 months prior to experiments. During acclimatization, fish were kept in tanks with running water. Water temperature was ~28°C and fish were reared at 12:12 h light: dark cycle. Fish were fed twice a day with commercial feed and wild type *W. globosa*. The animal studies were approved by the Animal Care and Use Committee of the Institute of Hydrobiology, Chinese Academy of Sciences (approval ID Keshuizhuan Y73Z061).

### Oral Vaccination and Sampling

**Figure 2:**
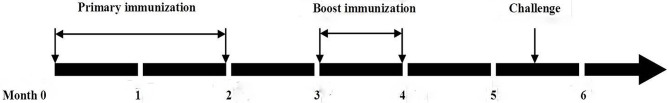
Design of oral immunization assay of zebrafish.

From the acclimatized fish, 270 apparently healthy (no clinical signs) individuals were randomly divided into three groups at a density of 30 fish/tank. A description of oral immunization and challenge infection is shown in Figure 2. The fresh biomass of wild type and transgenic *W. globosa* were used for the vaccination experiment. The fish in group 1 were fed with commercial feed twice a day during the experimental period and it was used as the control group. The fish in group 3 were immunized (primary immunization) by feeding with transgenic *W. globosa* and commercial feed twice a day for the period of 2 months. Then booster vaccination was performed for another month after 1 month interval of primary immunization. The fish in group 2 were fed with wild type untransformed *W. globosa* and commercial feed according to the schedule described in Figure 2. Oral vaccination experiments were performed in biological triplicates. Blood was collected from vaccinated and unvaccinated fish (from five fish at each time) at 5, 6, 7, 8, 9, and 10 weeks after booster vaccination. Blood (5 μl/fish) was collected from the caudal vein of fish by placing a capillary tube through the caudal fin cut. The blood was allowed to clot by keeping at room temperature for 2 h and then at 4°C overnight. Serum was collected after centrifugation at 750 × g for 10 min and was stored at −80°C for ELISA.

### Experimental Challenge and Calculation of Relative Percent Survival (RPS)

Six weeks post-vaccination, 30 fish from each group (group 1, 2, and 3) were anesthetized by immersing in tricaine methane sulfonate (MS-222) solution and challenged by intraperitoneal inoculation with 10 μl of 3.25 × 10^8^ cfu/ml cell suspension. Thirty fish were challenged by intraperitoneal inoculation with 10 μl PBS as control. Bacterial suspension was prepared as follows: virulent *Vibrio alginolyticus* was cultured at 28°C in TSB medium for 18 h, harvested by centrifugation at 4,000 g for 10 min and washed with PBS three times. Bacteria were suspended in 0.01 M PBS (pH 7.4) and adjusted to the concentration of 3.25 × 10^10^ cells/ml. In a preliminary assay, the median lethal dose (LD_50_) was determined to be 6.5 × 10^7^ CFU per fish. Fish from vaccinated and control groups were challenged using five times their lethal dose (LD_50_) (12). Fish mortality was monitored daily for 14 days, and dead fish were removed on a daily basis. The relative percent survival (RPS) was calculated by the following formula: RPS = [1− (% mortality of vaccinated fish/% mortality of control fish)] × 100%.

### Indirect ELISA for Detection of Antigen-Specific Serum Antibody

To analyze the protein by a more sensitive method for the detection of immunogenic epitopes of expressed protein, antibody titer was determined from the immunized fish by sandwich ELISA. Briefly, the sera of zebrafish were serially diluted in carbonate/bicarbonate buffer, pH 7.4 (1:1, v/v) and added in triplicate to wells of the plates. The plates were incubated overnight at 4°C. The remaining binding sites were blocked 1 h at 37°C with PBS-TS. Then 100 μl purified LamB (0.5 μg/well) was added to the plates. After incubation at 37°C for 2 h and washing three times with PBS-T, rabbit anti-LamB sera (1:1000 diluted in PBS-TS) were added to the plates. The plates were incubated and washed as above. HRP conjugated with goat-anti rabbit IgG (1:1000 diluted in PBS-TS) was added and incubated for another 1 h at 37°C and was then reacted with o-Phenylenediamine (OPD) substrate (Beyotime) for 10 min. Finally, the reaction was blocked by 2M H_2_SO_4_, and the absorbance of each and every well was measured at 492 nm with the help of Bio-Rad iMark micro-plate reader. The student's *t*-test was used to conduct significant tests.

## Results

### Amplification of Gene of Interest

From the genomic DNA of pathogenic *V. alginolyticus*, we successfully amplified the 1,200 bp full-length ORF of antigenic *LamB* gene. The deduced amino acid sequence of amplified gene consists of 447 amino acid residues. Gene was amplified using degenerate *LamB* primers as described by Lun et al. (12). According to Lun et al. (12), the annealing temperature of the PCR reaction was 58°C. However, though we were unable to amplify the *LamB* gene at this temperature, we were able to successfully amplify it at an annealing temperature of 48°C. The gene sequence was subjected to the homology search BLASTn in NCBI (http://www.ncbi.nlm.nih.gov/). Results showed 99% similarity with the *LamB* gene of *V. alginolyticus* (Accession no. EU625279). With this finding, the gene amplified was confirmed as the *LamB* gene of *V. alginolyticus* and it was used to construct the expression vector (pMYC-*LamB*) to transform antigenic *LamB* gene into *Wolffia* plant for antigenic protein expression trials.

### Molecular Characterization of Transgenic Plants

**Figure 3:**
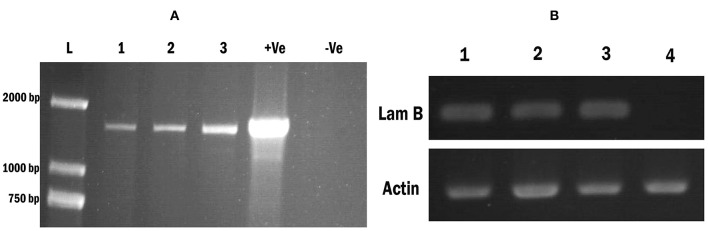
Screening transgenic *Wolffia* and confirmation of the modified *LamB* gene integration. **(A)** PCR amplification of modified *LamB* gene from *Wolffia* transformant. L: 2,000 bp ladder, Lane 1–3: contained 3 independent *LamB* integrated *Wolffia* lines. Lane 4: (Marked as +ve) contains plasmid extract containing LamB construct and it was used as the positive control. Lane 5: (marked –ve) contains genomic DNA from wild plants. **(B)** RT-PCR for the *LamB* in *Wolffia* transformant. An *Actin* gene was used as the internal control. Lanes 1–3: Putative *Wolffia* transformants, Lanes 4: Wild type plants.

We obtained hygromycin-resistant transgenic *Wolffia* fronds after 7–8 weeks of cultivation on SH medium in the presence of 5.0 mg l^−1^ hygromycin (Hyg). In total, 8 independent hygromycin-resistant transgenic *Wolffia* lines with *LamB* gene construct were identified and those were confirmed by PCR conducted with *LamB* gene specific primers (Figure 3A). To confirm that the *LamB* gene was correctly transcribed, the transgenic *Wolffia* were characterized in more detail by RT-PCR with *LamB* gene specific primers. PCR products of the expected size corresponding to the specific primers were detected in the transgenic *Wolffia*, whereas no amplified PCR product was detected in non-transformed wild-type *Wolffia* (Figure 3B). The *Actin* gene was used as an internal control. All transgenic *Wolffia* lines we tested were positive for the LamB transcript (Figure 3B).

### Immunoblot Detection of LamB Protein in Transgenic *W. globosa*

**Figure 4:**
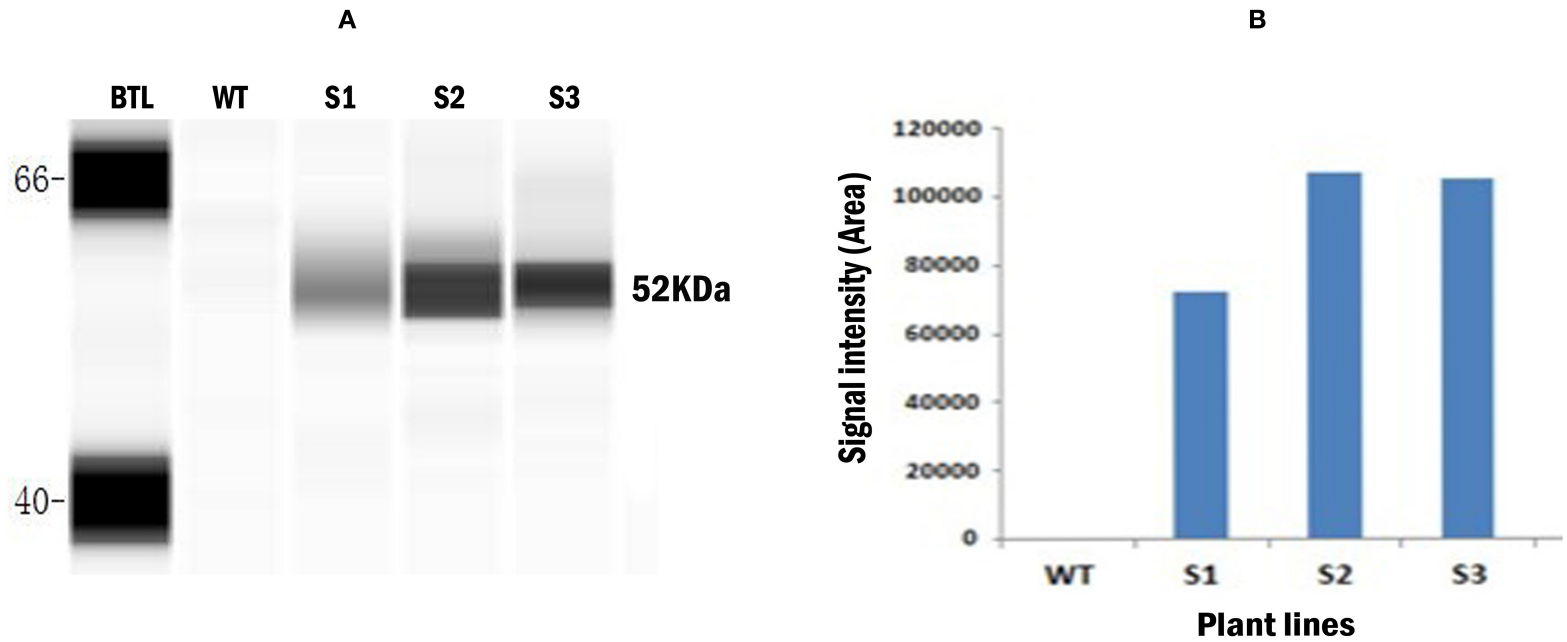
Capillary electrophoresis (CE) based Western blot assay conducted for detection of recombinant *LamB* protein in protein extracts of transgenic *Wolffia*. **(A)** Gel-like image viewed from Western blot assay. Left to right: BTL: Biotinylated ladder WT: Un-transformed wild type plant, S1–S3: Transformed plants. Anti- c- myc mouse monoclonal antibody was used as a primary antibody and the HRP-conjugated mouse anti-mouse IgG antibody used as a secondary antibody. **(B)** Signal intensity (area) shown in Capillary electrophoresis (CE) based Western blot assay for detection of recombinant LamB protein in protein extracts from the fronds of wild type and transformed *Wolffia*. WT - Wild type *Wolffia*, S1–S3 transgenic *Wolffia* lines.

CE based western blot assay was conducted with the total soluble protein extracted from the transgenic *Wolffia* to confirm the expression of recombinant *LamB* protein. Probing blots with mouse anti-myc primary antibody revealed full-length 52-kDa protein (Figure 4A). This product is consistent with the predicted size of fully intact recombinant LamB protein. The total soluble protein from wild-type plants did not show any band, indicating that mouse anti-myc antibody did not cross-react with any plant proteins in the crude extract. This confirmed the production of recombinant LamB antigenic protein in transformed *W. globosa*. The signal intensity (area) of CE based Western blot assay corresponding to the expression level of recombinant protein varied between three transgenic lines, indicating the different levels of protein expression in transgenic *Wolffia*. Figure 4B shows the different expression levels (signal intensity/area) obtained for three transgenic lines. The highest level of signals was detected in the S2 transgenic line and signal from S1 was comparatively weak according to the CE based western blot.

### Cross-Protective Analysis of LamB Recombinant Protein Produced in Transgenic *Wolffia*

Zebrafish were orally immunized with transgenic *Wolffia* and subsequently challenged with the pathogenic *V. alginolyticus* to evaluate the cross-protective potential of LamB recombinant protein produced in transgenic *Wolffia*. Fish mortality rate and sandwich ELISA were determined to evaluate the efficacy of this oral vaccine. Cumulative mortality of challenged zebrafish is shown in Table 2. Mortality rate of LamB vaccinated zebrafish was significantly lower compared to the control group (*P* < 0.05). The RPS of LamB vaccinated group was 63.3%. These results confirmed the protective capacity of LamB vaccinated zebrafish against *V. alginolyticus*. However, the antibodies against LamB protein in the sera of vaccinated fish were not detected by sandwich ELISA.

**Table 2:** Cumulative mortality of challenged zebrafish after vaccination by *V. alginolyticus* ECGY0608.

**Groups**	**Feed details**	**Cumulative percent mortality (Dead fish/total fish)**
1	Normal diet feed	100% (30/30)
2	50% normal diet + 50% wild type *Wolffia*	93.3% (28/30)
3	50% normal diet + 50% transgenic *Wolffia*	36.7% (11/30)

## Discussion

Among members of the duckweed family, *Wolffia* has been identified as a valuable bioreactor for foreign protein production (19). Lack of an efficient gene transformation protocol has limited the utilization of this valuable plant as a bio-manufacturing platform. To fulfill this gap in our previous work, we developed an efficient gene transformation protocol for *W. globosa*, the *Wolffia* species available in China (15). Use of antibiotics to control vibriosis in aquaculture causes serious health and environmental issues (20). Immunoprophylaxis would be a promising tool for effective control of this disease in a more effective and economical manner. Thus, as the second phase of our study, we attempted to demonstrate bioencapsulation of vaccinogenic outer membrane protein of vibrio in *Wolffia* for oral delivery against fish vibriosis.

With the morphological and functional differences investigated within the fish intestine, its second segment was identified as the main place of antigen uptake (5, 6). Studies conducted by Rombout et al. (21) and Firdaus (22) using fish with formalin killed *V. anguillarum* and feed-based adjuvant vaccine of *Streptococcus agalactiae* have proven that oral vaccination in fish could elicit certain levels of humoral and mucosal immunities following the development of gut-associated lymphoid tissue (GALT) (23). Thus, oral vaccines will be effective immune stimulators only if the antigenic substances reach the correct inductive sites in the fish gut. Accordingly, to achieve highly efficient edible vaccines, special techniques need to be developed to prevent antigenic substances from biodegradation in the proteolytic environment of the fish foregut till it reaches the local immune system.

Encapsulating vaccinogenic components in edible plants has become popular due to a number of advantages such as low production cost, direct administration by feeding without a needle and syringe and possible long-term storage without special storage facilities. Until now, many successful oral vaccines by expressing recombinant proteins in edible plants with higher expression level have been published for human diseases but for the fish diseases it is limited. In these studies, producing vaccine antigens in edible plants, including tomato, potato, lettuce, or rice has been identified as a viable approach for expressing mucosal vaccines (23–26). In particular, Shina et al. (27) has reported expression of a vaccinogenic recombinant major capsid protein (rMCP) of rock bream iridovirus in transgenic rice callus against iridovirus of fish. In this study oral immunization of Rock bream with rMCP in lyophilized rice callus powder has elicit the intestinal mucosal immunity for protection against iridovirus infection. This study suggests that oral administration of plant expressed vaccine antigen as a useful method to implement a vaccine program against fish diseases.

Recently duckweeds have been identified as a more efficient bio-manufacturing platform to express heterologous proteins. However few reports on utilizing duckweeds for protein expression can be seen (28–30) and *Lemna* was the commonly used species. Single attempt on utilizing *Wolffia* to express human granulocyte colony-stimulating factor (hG-CSF) by nuclear-transformation at the level of 0.002–0.2% has been reported (31). But expressing vaccine antigens has not been reported.

Outer membrane proteins (OMP) of gram negative bacteria are identified as a type of vaccine candidate and used to formulate vaccine against bacterial pathogens. OMPs of bacteria are being localized at the exteriors of the cell surface and are targets for bactericidal and protective antibodies (32, 33). Thus, we focused on LamB OMP of *Vibrio* to be expressed in *W. globosa*, which has already been identified as a broad cross protective vaccine antigen against fish vibriosis (12). We constructed LamB expression vector and introduced *LamB* gene into the nuclear genome of *Wolffia* as an alternative approach to prevent fish vibriosis.

Development of effective plant based edible vaccines deals with the level of foreign protein expressed in transgenic plants. Higher protein expression is necessary to induce protective immune responses of host via oral routes of delivery. Tobacco cryptic constitutive promoter (tCUP) is a translational enhancer sequence used for the expression vectors and it is being effective across a wide range of plant species, including monocots, dicots, or gymnosperms and a wide range of other organisms such as yeast (34). It can be combined with other promoters, such as CaMV 35S promoter, to elevate their activity further (34, 35). Thus, for our LamB expression construct, we used tCUP translational enhancer sequence to achieve higher expression of heterologous LamB protein in *Wolffia*. Moreover, signal peptides and endoplasmic reticulum (ER) retention signals are often used in expression vectors for correct compartmentalization and retention of the heterologous protein in plant ER (36–39). This minimizes the modifications happening to the glycoproteins in Golgi apparatus (40). Thus, we used PR1b signal peptide sequence and ER retention signal KDEL for our construct to direct and keep the homologous LamB protein in ER.

Finally using the pMYC-*LamB* construct and the *Agrobacterium*-mediated stable nuclear transformation protocol we developed, we successfully expressed the homologous *LamB* antigenic gene in *Wolffia*, and regeneration of transgenic *Wolffia* occurred at a satisfactory level. The PCR and transcription analysis conducted with the transgenic *Wolffia* indicated that the foreign genes have been transcribed correctly. All transgenic *Wolffia* lines were phenotypically indistinguishable from wild type control fronds. The development and growth rate of these transgenic *Wolffia* did not differ from the corresponding characteristics of the non-transformed control fronds. This indicates that the expression of the foreign protein has no effect on the growth and development of transgenic *Wolffia*. Thus, we successfully utilized this less/under exploited plant species, *W. globosa* as a novel vaccine expression platform to express the antigenic *LamB* against vibriosis. As an edible aquatic plant, this antigenic protein expressed transgenic *Wolffia* can be used to vaccinate the herbivorous fish through direct feeding without extraction and/or purification of the active constituent. To vaccinate the carnivorous fish, transgenic *Wolffia* can be fed with meat or commercial feeds.

The signal intensity (area) of CE based western blot assay corresponding to the expression level of recombinant LamB protein varied between three independent transgenic *Wolffia* lines. This indicates that the protein expression in the three transgenic lines is variable. This variation may be due to differences in the location/integration site of the newly introduced gene, the copy number of the T-DNA integrated and/or T-DNA organization (41, 42), as well as epigenetic effects (43). However, in our study we were unable to quantify the LamB protein accumulation in transgenic *Wolffia* due to the unavailability of protein standards.

The biological activity of recombinant LamB protein was assessed through bacterial challenge followed with an immunological assay. According to our findings the protective capacity of recombinant LamB against pathogenic *V. alginolyticus* in immunized fish were significant. This result confirmed the protective capacity of recombinant LamB against *V. alginolyticus* in fish. Even though the challenge test clearly indicates the protective capacity of transgenic *Wolffia*, with the ELISA we were unable to detect the antibodies against LamB in the sera of vaccinated fish. Some identified reasons for this type of erroneous results are competitive inhibition of test antibodies by relevant antibodies present in animal serum, denaturation of enzymes conjugated to detection antibodies, change in antibody specificity due to antigenic drift, sampling time and condition of the fish (44, 45).

In conclusion, we have successfully expressed antigenic LamB outer membrane protein of *Vibrio* in transgenic *Wolffia* against fish vibriosis and the successful nuclear transformation of LamB fusion protein with their functional properties is further demonstrated. Confirmation on its immunogenic property needs to be verified further by immunological studies to accomplish its immunostimulant property. However, our study opens the way to utilize the smallest flowering plant, *Wolffia*, for the development of low cost, easily deliverable and effective subunit vaccines.

## Data Availability Statement

All datasets generated for this study are included in the article/supplementary material.

## Ethics Statement

The animal study was reviewed and approved by Animal Care and Use Committee of the Institute of Hydrobiology, Chinese Academy of Sciences (approval ID Keshuizhuan Y73Z061).

## Author Contributions

HH and PH planned and designed the research. PH, ZS, JY, and XZ performed experiments and data analysis. PH, ZS, and HH wrote the manuscript. All authors contributed to manuscript revision and read and approved the submitted version.

## Conflict of Interest

The authors declare that the research was conducted in the absence of any commercial or financial relationships that could be construed as a potential conflict of interest.
